# Supplementary material for: Natural soundscapes enhance mood recovery amid anthropogenic noise pollution
Source: PLoS One. 2024 Nov 27;19(11):e0311487. doi: 10.1371/journal.pone.0311487 (PMC11602051; doi:10.1371/journal.pone.0311487)
Supplement: S1 Doc — (DOCX) [file pone.0311487.s001.docx]

**S1 Document.** Survey example extracted from Qualtrics software

Responding to a noisy world: a soundscape experiment

Start of Block: Default Question Block

Start

**Responding to a noisy world: a soundscape experiment**

 Thank you for taking the time to participate in this experiment.

 Before you start the experiment, we need you to read the following before consenting to take part: 
 
- Participant Information
 - Research Privacy Notice
 - Consent form

 **Click the arrow to proceed to the Participant Information Page.**

| Page Break |  |
| --- | --- |

Part_Info

**Participant Information**

***Participant info provided to participants-***

***Please click the arrow on the bottom right hand side to read the Research Privacy Notice on the next page.***
  

| Page Break |  |
| --- | --- |

Privacy Research

Privacy Notice for Research Participants

***Please click the arrow at the bottom right hand side of the page to go to the Consent Form.***

| Page Break |  |
| --- | --- |

Consent

**Consent form provided to participants**
  
 ***Type your signature (full name) in the box below to consent to taking part and click the arrow on the bottom right hand side to proceed to the experiment.***

________________________________________________________________

| Page Break |  |
| --- | --- |

Info

**Responding to a noisy world: a soundscapes experiment**
**Before you start**  

Please stay in one place whilst carrying out this experiment. Do not carry out this experiment whilst walking or travelling on public transport. Do not carry out this experiment under the influence of alcohol or recreational drugs, as this may influence the outcomes of the experiment.

Please close all other programs and/or tabs open on your computer (e.g. emails, social media, work), turn your phone on silent and turn off any other notifications that might disturb you during the experiment.

Noise cancelling (over the ear) headphones are preferable for use in this experiment, but if you do not have these, you can use ear buds (in the ear) or computer/laptop speakers.

The experiment should take around 30 minutes to an hour to complete and you should finish it in one sitting. Please make sure you have enough time to finish the entire experiment before starting.

Once you have answered a question, please do not navigate back and change you answers. This will invalidate the results.

Before you start the experiment, you may wish to have a pen and paper ready, to be able to note down your answers to some of the questions.

*You will be told when to use the pen and paper and you will see this sign.*

| Page Break |  |
| --- | --- |

STAI_trait

**How do you feel generally?**

 *Please answer some questions about how you feel generally.*

A number of statements which people have used to describe themselves are given below. 
Read each statement on the left hand side and in each case click the ‘slider’ to move it and place it underneath the word(s) at the top to indicate how you *generally* feel.  **There are no right or wrong answers**.

 Do not spend too much time on any one statement but give the answer which seems to describe how you generally feel.

 *Once you have answered all of the slider questions, please click the arrow at the bottom right hand side to proceed to the rest of the experiment.*

|  | **Almost never** | **Sometimes** | **Often** | **Almost always** |
| --- | --- | --- | --- | --- |

|  | 1 | 2 | 3 | 4 |
| --- | --- | --- | --- | --- |

| I am "calm, cool and collected" () | 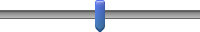 |
| --- | --- |
| I worry too much over something that really doesn’t matter () | 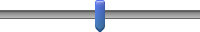 |
| I feel secure () | 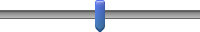 |
| I get in a state of tension or turmoil as I think over my recent concerns and interests () | 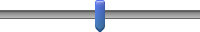 |
| I feel nervous and restless () | 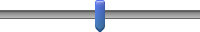 |
| I make decisions easily () | 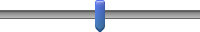 |

| Page Break |  |
| --- | --- |

Sound_test

**Sound Test**

 Please place your **headphones on** and click on the 'Test' link below to do a **sound test**. 
 You will be taken to another tab where some test sounds of people talking will play.
 *Adjust your headphones to a level that is comfortable, but loud enough to immerse yourself in the sounds.*
   *Once you have finished the sound test, please click back on the survey tab and navigate to the next bit of the survey using the bottom right hand side arrow button.*   *You do not have to listen to the whole sound clip, just enough to adjust your headphones/speakers to a comfortable level.*

 Test

End of Block: Default Question Block

Start of Block: Block 1

Stress_1 Please click on the link to watch the video below and note down your answers to questions. 

 *A new tab will open, please watch the video* ***only once*** *and do not stop the video once it has started.

 Once the video has finished, please click back on the survey tab.*

   
Click for video

 *If you have watched the video please click on the arrow at the bottom right hand side to immediately complete the questions on the next page*.

| Page Break |  |
| --- | --- |

UWIST_1

**H**ow do you feel now?
  
A number of words which people have used to describe themselves are given below. 

Read each word on the left hand side and in each case click the ‘slider’ to move it and place it underneath the word at the top to indicate how you feel right now, that is, at this moment. 

There are no right or wrong answers.

*Please proceed to the next slider task immediately after completing the one below. Do not navigate back.*

|  | **Definitely not** | **Not much** | **Quite** | **Definitely** |
| --- | --- | --- | --- | --- |

|  | 1 | 2 | 3 | 4 |
| --- | --- | --- | --- | --- |

| Relaxed () | 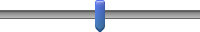 |
| --- | --- |
| Sad () | 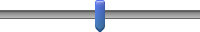 |
| Happy () | 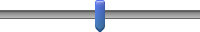 |
| Nervous () | 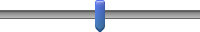 |

STAI_1

How do you feel now?
 

Read each statement on the left hand side and in each case click the ‘slider’ to move it and place it underneath the word at the top to indicate how you feel *right now*, that is, at this moment. 


There are no right or wrong answers.


Do not spend too much time on any one statement but give the answer which seems to describe your present feelings best.
 
*Once you have answered all of the slider questions, please click the arrow at the bottom right hand side to proceed to the rest of the experiment.* 

|  | **Not at all** | **Somewhat** | **Moderately so** | **Very much so** |
| --- | --- | --- | --- | --- |

|  | 1 | 2 | 3 | 4 |
| --- | --- | --- | --- | --- |

| I feel calm () | 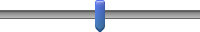 |
| --- | --- |
| I am tense () | 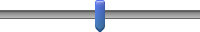 |
| I feel at ease () | 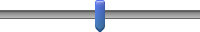 |
| I feel nervous () | 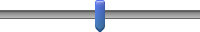 |
| I am relaxed () | 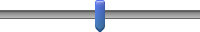 |
| I am worried () | 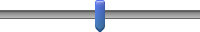 |

| Page Break |  |
| --- | --- |

Sound_1 Please click on the link below and listen to the soundscape.

 *A new tab will open and the soundscape will begin to play.
 The length of the soundscape is 3 minutes.
 After you have listened to the soundscape, please click back on the survey tab and navigate to the next section of the experiment.*   You might want to close your eyes and immerse yourself in the sounds.    [Soundscape 1](https://uwe.eu.qualtrics.com/CP/File.php?F=F_bHfJuSlZrXw2JPn)
   *Please click the arrow on the bottom right hand side below to proceed to the next page to immediately complete the questions on the next page.*

| Page Break |  |
| --- | --- |

UWIST_2

How do you feel now?
A number of words which people have used to describe themselves are given below. 
 
Read each word on the left hand side and in each case click the ‘slider’ to move it and place it underneath the word at the top to indicate how you feel right now, that is, at this moment. 

There are no right or wrong answers.
  
*Please proceed to the next slider task immediately after completing the one below. Do not navigate back.*

|  | **Definitely not** | **Not much** | **Quite** | **Definitely** |
| --- | --- | --- | --- | --- |

|  | 1 | 2 | 3 | 4 |
| --- | --- | --- | --- | --- |

| Relaxed () | 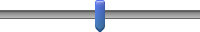 |
| --- | --- |
| Sad () | 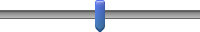 |
| Happy () | 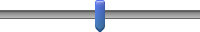 |
| Nervous () | 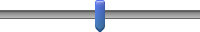 |

STAI_2
How do you feel now?
Read each statement on the left hand side and in each case click the ‘slider’ to move it and place it underneath the word at the top to indicate how you feel *right now*, that is, at this moment. 

There are no right or wrong answers.

Do not spend too much time on any one statement but give the answer which seems to describe your present feelings best.
 
*Once you have answered all of the slider questions, please click the arrow at the bottom right hand side to proceed to the rest of the experiment.* 

|  | **Not at all** | **Somewhat** | **Moderately so** | **Very much so** |
| --- | --- | --- | --- | --- |

|  | 1 | 2 | 3 | 4 |
| --- | --- | --- | --- | --- |

| I feel calm () | 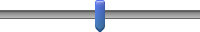 |
| --- | --- |
| I am tense () | 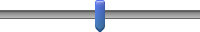 |
| I feel at ease () | 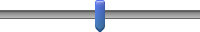 |
| I feel nervous () | 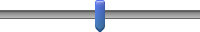 |
| I am relaxed () | 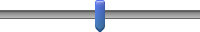 |
| I am worried () | 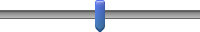 |

| Page Break |  |
| --- | --- |

*Repeat process stressor then soundscape two more times, with soundscape randomized*

Start of Block: Block 3

Age

What is your age?

________________________________________________________________

Gender

What is your gender?

- Male (1)
- Female (2)
- Non-binary / third gender (3)
- Prefer not to say (4)

prefs

To what extent do you have a preference for urban and natural environments?

- Strong preference for natural environments (1)
- Slight preference for natural envirnoments (2)
- No preference (3)
- Slight preference for urban environments (4)
- Strong preference for urban environments (5)

sounds

During your daily life, to what extent do you notice sounds in your environment, both natural and manmade?

- Very often (1)
- Often (2)
- Not sure (3)
- Sometimes (4)
- Never (5)

live

What sort of environment do you currently work and live in?

*Select the answer for* ***urban environment*** *if you live/work in a large town or a city. Select the answer for* ***rural environment*** *if you live/work in a small town or village in the countryside or a more remote rural area. Select semi-rural if you live/work in a small town in the countryside.*

- Currently live in urban environment (large town or city) and work in an urban environment (1)
- Currently live in a rural environment (countryside) and work in a rural environment (2)
- Currently live in urban environment (large town or city) and work in rural environment (countryside) (3)
- Currently live in a rural environment (countryside) and work in an urban environment (large town or city) (4)
- Currently live in a semi-rural environment and work in an urban environment (large town or city) (6)
- Currently live in a semi-rural environment and work in an rural environment (countryside) (7)
- Currently live in urban environment (large town or city) and work in an semi-rural environment (8)
- Currently live in a rural environment (countryside) and work in a semi-rural environment (9)
- Currently live in a semi-rural environment and work in an semi-rural environment (10)

grow_up

What sort of environment did you grow up in?

*Select* ***urban environment*** *if you grew up in a large town or city and* ***rural environment*** *if you grew up in a village, or a more rural area in the countryside. Select****semi-rural****if you grew up in a small town in the countryside.*

- Grew up in an urban environment (large town or city) (1)
- Grew up in a rural environment (countryside) (2)
- Grew up in a semi-rural environment (small town in countryside) (3)

phobias Do you have any phobias? Please select all that apply below:

- spiders (1)
- cats (2)
- dogs (3)
- birds (4)
- bats (5)
- snakes (6)

ethnicity What is your ethnicity? Please select the category below that defines your ethnicity.

▼ White (English, Welsh, Scottish, Northern Irish or British / Irish / Gypsy or Irish Traveller / Any other White background) (1) ... Not stated (6)

headphones How did you listen to the soundscapes in the experiment today?

▼ Noise cancelling headphones or over the ear headphones (1) ... Other (4)

comments Any other comments

________________________________________________________________

________________________________________________________________

________________________________________________________________

________________________________________________________________

________________________________________________________________

| Page Break |  |
| --- | --- |

Thanks

**Thank you for taking part!**

 **IMPORTANT!**
 ***Please click the arrow at the bottom right hand side of the page, to end the survey.***
***(If you do not click the final arrow, your data will not be saved)***

End of Block: Block 3
