# Supplementary material for: Natural soundscapes enhance mood recovery amid anthropogenic noise pollution
Source: PLoS One. 2024 Nov 27;19(11):e0311487. doi: 10.1371/journal.pone.0311487 (PMC11602051; doi:10.1371/journal.pone.0311487)
Supplement: S1 Fig — (DOCX) [file pone.0311487.s002.docx]

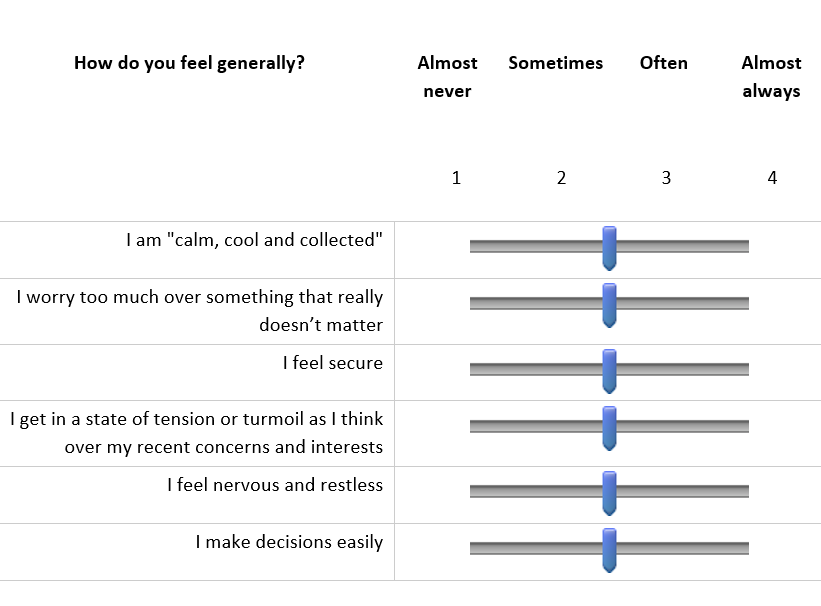


**Figure S1.** Example of 4-point Likert scale presented to participants before the experiment started to get a measure of ***trait*** anxiety, or general mood. Including six mood items, adapted from a validated short form of the State-Trait Anxiety Inventory (STAI) scale (Spielberger et al. 1970; Fioravanti-Bastos et al. 2011).
