# Supplementary material for: Natural soundscapes enhance mood recovery amid anthropogenic noise pollution
Source: PLoS One. 2024 Nov 27;19(11):e0311487. doi: 10.1371/journal.pone.0311487 (PMC11602051; doi:10.1371/journal.pone.0311487)
Supplement: S2 Fig — (DOCX) [file pone.0311487.s003.docx]

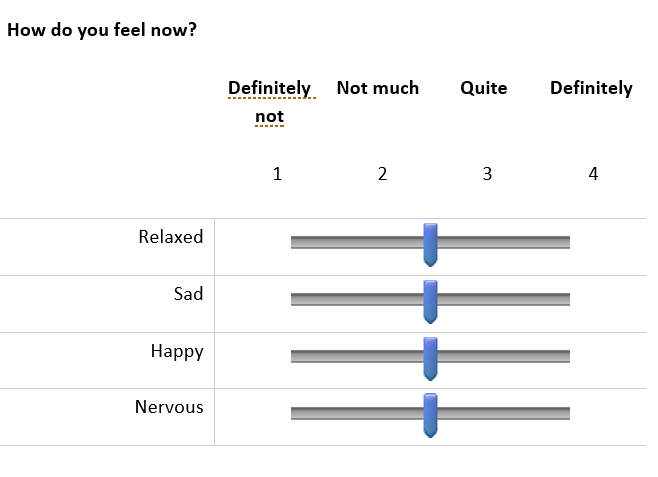


**Figure S2.** Example of 4-point Likert scale presented to participants after each stressor video and soundscape file. Including four mood ***state*** items, designed to measure current mood in terms of subjective stress and hedonic tone using a short form of the University of Wales Institute of Science and Technology Mood Adjective Checklist (UWIST MACL) (Matthews et al. 1990).
