# Supplementary material for: Natural soundscapes enhance mood recovery amid anthropogenic noise pollution
Source: PLoS One. 2024 Nov 27;19(11):e0311487. doi: 10.1371/journal.pone.0311487 (PMC11602051; doi:10.1371/journal.pone.0311487)
Supplement: S1 Table — (DOCX) [file pone.0311487.s005.docx]

**Table S1.** Demographic data for all participants (n=68), including mean and s.d. for continuous variables and % for factor variables.

| \| **Demographics** \| **Demographic level** \| **Mean (s.d.)/%** \| \| --- \| --- \| --- \| \| **Age** \| Age \| 20.88 (3.58) \| \| **Baseline anxiety level** \| STAI-T score \| 14.50 (3.32) \| \| **Sex** \| Male \| 29.41 \| \|  \| Female \| 69.12 \| \|  \| Non-binary \| 1.47 \| \| **Environment Preferences** \| Preference natural (slight) \| 32.35 \| \|  \| Preference natural (strong) \| 33.82 \| \|  \| Preference urban (slight) \| 7.35 \| \|  \| Preference urban (strong) \| 5.88 \| \|  \| No Preference environ \| 20.59 \| \| **Notice sounds in environment?** \| Not sure Notice sounds \| 8.82 \| \|  \| Often Notice sounds \| 30.88 \| \|  \| Sometimes Notice sounds \| 22.06 \| \|  \| Very often Notice sounds \| 38.24 \| \| **Live and work environment** \| Live rural work rural \| 2.94 \| \|  \| Live rural work urban \| 2.94 \| \|  \| Live semi-rural work rural \| 1.47 \| \|  \| Live semi-rural work semi-rural \| 14.71 \| \|  \| Live semi-rural work urban \| 7.35 \| \|  \| Live urban work rural \| 2.94 \| \|  \| Live urban work semi-rural \| 1.47 \| \|  \| Live urban work urban \| 66.18 \| \| **Listening device** \| Computer/laptop speakers \| 22.06 \| \|  \| In-ear headphones \| 41.18 \| \|  \| Over ear headphones \| 36.76 \| \| **Grow up** \| Rural \| 16.18 \| \|  \| Semi-rural \| 39.71 \| \|  \| Urban \| 44.12 \| \| **Phobias** \| Birds \| 1.47 \| \|  \| Other \| 60.29 \| \| **Ethnicity** \| White/British \| 80.88 \| \|  \| BME \| 17.65 \| \|  \| Other* \| 1.47 \| \| **Course studying** \| Psychology \| 51.47 \| \|  \| Other \| 48.53 \| \| **Course level** \| 1st year undergraduate \| 44.12 \| \|  \| 2nd year undergraduate \| 38.24 \| \|  \| Postgraduate (masters) \| 1.47 \| |  |  |
| --- | --- | --- | --- | --- | --- | --- | --- | --- | --- | --- | --- | --- | --- | --- | --- | --- | --- | --- | --- | --- | --- | --- | --- | --- | --- | --- | --- | --- | --- | --- | --- | --- | --- | --- | --- | --- | --- | --- | --- | --- | --- | --- | --- | --- | --- | --- | --- | --- | --- | --- | --- | --- | --- | --- | --- | --- | --- | --- | --- | --- | --- | --- | --- | --- | --- | --- | --- | --- | --- | --- | --- | --- | --- | --- | --- | --- | --- | --- | --- | --- | --- | --- | --- | --- | --- | --- | --- | --- | --- | --- | --- | --- | --- | --- | --- | --- | --- | --- | --- | --- | --- | --- | --- | --- | --- | --- | --- | --- | --- | --- | --- | --- | --- | --- | --- | --- | --- | --- | --- |
| *Biological sciences, biomedical sciences, criminology, criminology and sociology/psychology, Ecology & wildlife conservation, environmental science, forensic science, foundation biology, MSc public health, psychology with criminology, social sciences |  |  |
