# Supplementary material for: Natural soundscapes enhance mood recovery amid anthropogenic noise pollution
Source: PLoS One. 2024 Nov 27;19(11):e0311487. doi: 10.1371/journal.pone.0311487 (PMC11602051; doi:10.1371/journal.pone.0311487)
Supplement: S2 Table — (DOCX) [file pone.0311487.s006.docx]

| **Table S2.** Final GLMM statistics for subjective measures UWIST MACL stress and hedonic tone (hedtone) and STAI-S anxiety scores, including significant fixed effects soundscape treatment and STAI-T scores for all three subjective measures and stressor treatment for hedtone.   \| **Model** \| **Model terms** \| **Estimate** \| **s.d.** \| ***t*** \| \| --- \| --- \| --- \| --- \| --- \| \| **Stress** \| (Intercept) \| 2.10 \| 0.47 \| 4.50 \| \|  \| Bird + 20mph \| 0.25 \| 0.15 \| 1.65 \| \|  \| Bird + 40mph \| 0.51 \| 0.15 \| 3.39 \| \|  \| STAI-T \| 0.06 \| 0.03 \| 2.07 \| \|  \| Random effects \| Variance \| s.d. \| % total \| \|  \| Participants (N = 68) \| 0.45 \| 0.67 \| 36.47 \| \|  \| Residual \| 0.78 \| 0.89 \| 63.53 \| \|  \| No observations \| 204.00 \|  \|  \| \| **Hedtone** \| (Intercept) \| 7.65 \| 0.58 \| 13.27 \| \|  \| Bird + 20mph \| 0.02 \| 0.14 \| 0.18 \| \|  \| Bird + 40mph \| -0.29 \| 0.14 \| -2.10 \| \|  \| Stressor B \| -0.04 \| 0.14 \| -0.26 \| \|  \| Stressor C \| -0.31 \| 0.14 \| -2.29 \| \|  \| STAI_T \| -0.09 \| 0.04 \| -2.26 \| \|  \| Random effects \| Variance \| s.d. \| % total \| \|  \| Participants (N = 68) \| 0.86 \| 0.93 \| 58.06 \| \|  \| Residual \| 0.62 \| 0.79 \| 41.94 \| \|  \| No observations \| 204.00 \|  \|  \| \| **Anxiety** \| (Intercept) \| 6.41 \| 1.38 \| 4.66 \| \|  \| Bird + 20mph \| 1.29 \| 0.37 \| 3.49 \| \|  \| Bird + 40mph \| 1.75 \| 0.37 \| 4.71 \| \|  \| STAI-T \| 0.22 \| 0.09 \| 2.38 \| \|  \| Random effects \| Variance \| s.d. \| % total \| \|  \| Participants (N = 68) \| 4.67 \| 2.16 \| 49.90 \| \|  \| Residual \| 4.69 \| 2.17 \| 50.10 \| \|  \| No observations \| 204.00 \|  \|  \| |  |  |
| --- | --- | --- | --- | --- | --- | --- | --- | --- | --- | --- | --- | --- | --- | --- | --- | --- | --- | --- | --- | --- | --- | --- | --- | --- | --- | --- | --- | --- | --- | --- | --- | --- | --- | --- | --- | --- | --- | --- | --- | --- | --- | --- | --- | --- | --- | --- | --- | --- | --- | --- | --- | --- | --- | --- | --- | --- | --- | --- | --- | --- | --- | --- | --- | --- | --- | --- | --- | --- | --- | --- | --- | --- | --- | --- | --- | --- | --- | --- | --- | --- | --- | --- | --- | --- | --- | --- | --- | --- | --- | --- | --- | --- | --- | --- | --- | --- | --- | --- | --- | --- | --- | --- | --- | --- | --- | --- | --- | --- | --- | --- | --- | --- | --- | --- | --- | --- | --- | --- | --- | --- | --- | --- | --- | --- | --- | --- | --- | --- | --- | --- | --- | --- | --- | --- | --- | --- | --- |
|  |  |  |
|  |  |  |
|  |  |  |
|  |  |  |
|  |  |  |
|  |  |  |
|  |  |  |
|  |  |  |
|  |  |  |
|  |  |  |
|  |  |  |
|  |  |  |
|  |  |  |
