# Supplementary material for: Natural soundscapes enhance mood recovery amid anthropogenic noise pollution
Source: PLoS One. 2024 Nov 27;19(11):e0311487. doi: 10.1371/journal.pone.0311487 (PMC11602051; doi:10.1371/journal.pone.0311487)
Supplement: S3 Table — (DOCX) [file pone.0311487.s007.docx]

| **Table S3.** Model selection statistics for GLMM for three subjective measures, UWIST MACL stress and hedtone and STAI state anxiety, arranged by AICc weight (AICc Wt) and including number of model parameters (K), cumulative AICc weight (Cum. Wt). Final models in bold.   \| **Model** \| **Fixed effects** \| **Distribution** \| **K** \| **AICc** \| **ΔAICc** \| **AICc Wt** \| **Cum. Wt** \| \| --- \| --- \| --- \| --- \| --- \| --- \| --- \| --- \| \| **Stress** \| **Soundscape + STAI_T** \| **gaussian** \| **6** \| **617.48** \| **0.00** \| **0.70** \| **0.70** \| \|  \| STAI_T \| gaussian \| 4 \| 620.29 \| 2.80 \| 0.17 \| 0.87 \| \|  \| Soundscape + Stressor + STAI_T \| gaussian \| 7 \| 621.15 \| 3.70 \| 0.11 \| 0.98 \| \|  \| Soundscape*STAI_T \| gaussian \| 8 \| 625.51 \| 8.00 \| 0.01 \| 1.00 \| \|  \| Soundscape+Stressor+Age+STAI_T \| gaussian \| 8 \| 628.46 \| 11.00 \| 0.00 \| 1.00 \| \|  \| Soundscape+Stressor+Age+Gender+Pref_binary \| gaussian \| 10 \| 631.87 \| 14.40 \| <0.001 \| 1.00 \| \|  \| Soundscape+Stressor+Age+Gender+STAI_T \| gaussian \| 10 \| 632.19 \| 14.70 \| <0.001 \| 1.00 \| \|  \| Soundscape+Stressor+Age+Gender+Pref_binary+STAI_T \| gaussian \| 11 \| 635.32 \| 17.80 \| <0.001 \| 1.00 \| \| **Hedtone** \| Soundscape+STAI_T \| gaussian \| 6 \| 618.60 \| 0.00 \| 0.45 \| 0.45 \| \|  \| STAI_T+Stressor \| gaussian \| 6 \| 618.79 \| 0.20 \| 0.42 \| 0.87 \| \|  \| **Soundscape+Stressor+STAI_T** \| **gaussian** \| **8** \| **621.28** \| **2.70** \| **0.12** \| **0.99** \| \|  \| Soundscape+Stressor+Age+STAI_T \| gaussian \| 9 \| 628.21 \| 9.60 \| 0.00 \| 0.99 \| \|  \| Soundscape*Stressor+STAI_T \| gaussian \| 12 \| 628.68 \| 10.10 \| 0.00 \| 1.00 \| \|  \| Soundscape+Stressor+Age+Gender+Pref_binary \| gaussian \| 11 \| 629.44 \| 10.80 \| 0.00 \| 1.00 \| \|  \| Soundscape+Stressor+Age+Gender+STAI_T \| gaussian \| 11 \| 629.94 \| 11.30 \| 0.00 \| 1.00 \| \|  \| Soundscape+Stressor+Age+Gender+Pref_binary+STAI_T \| gaussian \| 12 \| 632.53 \| 13.90 \| <0.001 \| 1.00 \| \|  \| Soundscape*STAI_T+Stressor \| gaussian \| 10 \| 633.27 \| 14.70 \| <0.001 \| 1.00 \| \| **Anxiety** \| **Soundscape+STAI_T** \| **gaussian** \| **6** \| **1000.58** \| **0.00** \| **0.61** \| **0.61** \| \|  \| Soundscape+Stressor+STAI_T \| gaussian \| 7 \| 1001.85 \| 1.30 \| 0.32 \| 0.94 \| \|  \| Soundscape+Stressor+Age+STAI_T \| gaussian \| 8 \| 1007.02 \| 6.40 \| 0.02 \| 0.96 \| \|  \| Soundscape+Stressor+Age+Gender+STAI_T \| gaussian \| 10 \| 1007.70 \| 7.10 \| 0.02 \| 0.98 \| \|  \| Soundscape*STAI_T \| gaussian \| 8 \| 1008.29 \| 7.70 \| 0.01 \| 0.99 \| \|  \| Soundscape+Stressor+Age+Gender+Pref_binary+STAI_T \| gaussian \| 11 \| 1009.81 \| 9.20 \| 0.01 \| 1.00 \| \|  \| Soundscape+Stressor+Age+Gender+Pref_binary \| gaussian \| 10 \| 1010.65 \| 10.10 \| 0.00 \| 1.00 \| \|  \| model1f<-lmer(STAI_sound_reverse~STAI_T \| gaussian \| 4 \| 1018.10 \| 17.50 \| <0.001 \| 1.00 \| |  |  |
| --- | --- | --- | --- | --- | --- | --- | --- | --- | --- | --- | --- | --- | --- | --- | --- | --- | --- | --- | --- | --- | --- | --- | --- | --- | --- | --- | --- | --- | --- | --- | --- | --- | --- | --- | --- | --- | --- | --- | --- | --- | --- | --- | --- | --- | --- | --- | --- | --- | --- | --- | --- | --- | --- | --- | --- | --- | --- | --- | --- | --- | --- | --- | --- | --- | --- | --- | --- | --- | --- | --- | --- | --- | --- | --- | --- | --- | --- | --- | --- | --- | --- | --- | --- | --- | --- | --- | --- | --- | --- | --- | --- | --- | --- | --- | --- | --- | --- | --- | --- | --- | --- | --- | --- | --- | --- | --- | --- | --- | --- | --- | --- | --- | --- | --- | --- | --- | --- | --- | --- | --- | --- | --- | --- | --- | --- | --- | --- | --- | --- | --- | --- | --- | --- | --- | --- | --- | --- | --- | --- | --- | --- | --- | --- | --- | --- | --- | --- | --- | --- | --- | --- | --- | --- | --- | --- | --- | --- | --- | --- | --- | --- | --- | --- | --- | --- | --- | --- | --- | --- | --- | --- | --- | --- | --- | --- | --- | --- | --- | --- | --- | --- | --- | --- | --- | --- | --- | --- | --- | --- | --- | --- | --- | --- | --- | --- | --- | --- | --- | --- | --- | --- | --- | --- | --- | --- | --- | --- | --- | --- | --- |
